# CD3-CSPG4 bispecific T cell engager: A novel platform for malignant melanoma therapy

**DOI:** 10.1371/journal.pone.0357790

**Published:** 2026-10-05

**Authors:** Xue Lei, Weilan Liu, Zongnian Luo, Bangyong Wu, Yu Meng, Qiang Jiang

**Affiliations:** 1 Department of Laboratory Medicine, The People’s Hospital of Qiannan, Duyun, Guizhou, China; 2 Department of Dermatology, The People’s Hospital of Qiannan, Duyun, Guizhou, China; University of Health Sciences Lahore, PAKISTAN

## Abstract

Malignant melanoma (MM) is a highly aggressive malignancy that readily metastasizes to lymph nodes and distant sites through hematogenous spread, leading to poor clinical outcomes. Therefore, there is an urgent need to develop novel therapeutic strategies. Chondroitin sulfate proteoglycan 4 (CSPG4) is one of the most frequently overexpressed proteins on malignant melanoma cells, whereas it is only expressed at very low levels in normal tissues. In this study, a novel bispecific T‑cell engager, CD3‑CSPG4/BiTE, was constructed by linking single‑chain antibodies directed against CSPG4 and CD3. In vitro assays assessed its simultaneous and specific binding to CSPG4 ⁺ melanoma cells and T cells, its capacity to drive T-cell proliferation and activation, and its ability to redirect T-cell cytotoxicity toward CSPG4 ⁺ targets. Additionally, in NOD/SCID mice bearing established human melanoma xenografts, the antitumor efficacy and systemic toxicity of the molecule were evaluated. CD3-CSPG4/BiTE was successfully generated and was shown to simultaneously bind CSPG4 ⁺ melanoma cells and T cells. Flow-cytometric analysis revealed that, compared with control constructs, CD3-CSPG4/BiTE markedly enhanced T-cell proliferation and activation (as evidenced by up-regulated CD25 and CD69) and effectively redirected T-cell–mediated lysis of CSPG4 ⁺ targets in vitro. In NOD/SCID mice bearing human melanoma xenografts, the BiTE significantly suppressed tumor growth without eliciting detectable toxicity in major organs. In summary, this study successfully developed a novel bispecific T-cell engager, CD3-CSPG4/BiTE, and demonstrated its potent antitumor activity against malignant melanoma both in vitro and in vivo. These findings provide a preclinical foundation for the clinical translation and further optimization of BiTE-based immunotherapy for melanoma.

## Background

Malignant melanoma (MM) is a highly aggressive malignancy characterized by early lymph node and hematogenous dissemination, leading to poor clinical outcomes. Its incidence has steadily increased: between 2003 and 2018, the age‑standardized clinical incidence rose by 23.9% in men and 27.3% in women [1], imposing a substantial economic and social burden. Prognosis is closely associated with tumor stage, with long‑term overall survival for patients with advanced disease remaining below 10% [2,3]. Current treatment primarily involves wide local excision, with resection margins determined by tumor thickness. Subsequent management is guided by pathological staging and, when indicated, sentinel lymph node biopsy, and may include surgery, immune checkpoint inhibitors, targeted therapy, or radiotherapy. For patients with resectable locoregional metastases, systemic agents may be administered as postoperative adjuvant therapy [4,5]. Among these, CAR‑T cell therapy, TCR‑T cell therapy, and oncolytic virotherapy have shown promising efficacy but are also associated with adverse events such as cytokine release syndrome. Therefore, there is an urgent need to develop targeted therapies with fewer side effects [6,7].

Bispecific T-cell engagers (BiTEs) represent a novel class of cancer immunotherapeutics. Through genetic engineering, an anti-CD3 single-chain variable fragment (scFv) derived from T cells is linked via a short peptide linker to an scFv directed against a tumor‑associated antigen. This construct redirects and activates polyclonal T cells, enabling them to recognize and efficiently eliminate tumor cells [8,9]. The physical linkage forces close apposition of T cells and tumor cells, triggering immediate T-cell activation and robust clonal expansion. Activated T cells release perforin, which polymerizes in the tumor cell membrane to form transmembrane pores, and granzymes, which enter through these pores and proteolytically degrade intracellular proteins, ultimately leading to tumor cell apoptosis [10,11]. BiTEs not only enable MHC‑independent activation of polyclonal T cells to eradicate cancer cells, but also, owing to their relatively small molecular size, penetrate solid tumors more efficiently and engage T cells with high avidity [12]. Clinically, blinatumomab is the first BiTE approved for cancer therapy. By simultaneously linking CD19-positive tumor cells to CD3-positive T cells via its anti-CD19 and anti-CD3 scFvs, it activates and expands T cells, directing them to selectively kill CD19-positive leukemic blasts. It is indicated for the treatment of patients with B-cell precursor acute lymphoblastic leukemia (B-ALL) [13,14]. These findings demonstrate that BiTE‑based immunotherapy is a feasible and viable strategy for cancer treatment.

Due to its high mutational burden, (MM) generates numerous tumor-associated antigens and neoantigens that can serve as immunotherapeutic targets. Chondroitin sulfate proteoglycan 4 (CSPG4), also known as melanoma-associated chondroitin sulfate proteoglycan (or neuron‑glial antigen-2 in rodents) [15], is a transmembrane protein whose expression has recently been detected in a variety of malignancies, including melanoma, glioma, triple-negative breast cancer, and pancreatic cancer, while remaining limited in normal tissues [16,17]. Consequently, CSPG4 has been proposed as a promising therapeutic target.

In summary, this project employs single‑chain antibodies directed against CSPG4 and CD3 to investigate their therapeutic potential against malignant melanoma. Through comprehensive evaluation of the antitumor efficacy of the novel CD3‑CSPG4/BiTE antibody construct both in vitro and in vivo, we aim to enhance its targeting specificity and therapeutic index for melanoma while minimizing adverse effects, thereby achieving more precise and effective cancer therapy.

## Materials and methods

### Construction of CD3-CSPG4/BiTE

In this study, the high-affinity anti-CD3 scFv gene (derived from the published patent CN1421459A) and the high-affinity anti-CSPG4 scFv gene (derived from the published patent US11725061 B2) were genetically linked via a flexible linker sequence using standard gene engineering techniques. Additionally, sequences encoding a His-tag and a Flag-tag were inserted to facilitate downstream purification and identification. The resulting recombinant construct was then cloned into the pET-30a(+) E. coli expression plasmid. Of note, the CD3-TTE/BiTE construct actually used in this study targets fibroblast activation protein (FAP) and was generated in our previous work. After IPTG induction, the protein was purified via His-tag affinity chromatography and verified by SDS-PAGE, followed by large-scale expression and purification for downstream experiments. The homology-modelling program SWISS-MODEL was used to generate the CD3-CSPG4/BiTE structure (designated the CD3-CSPG4/BiTE model); the modelling templates and sequences were derived from the PDB database.

### Cell cultures

The study selected CSPG4 high-expressing melanoma cells (A375, A2058) and CSPG4 low-expressing melanoma (SBCL-2) cell lines, all cells were preserved in our laboratory. The above cells were cultured in high glucose DMEM complete cell culture medium containing 10% Fetal bovine serum (FBS) and 1% penicillin and streptomycin mixture in a constant temperature incubator containing 5% CO2 and 37℃.

This study collected human peripheral blood from healthy adult volunteers between 01/06/2024 and 30/07/2025, with 40 mL of blood drawn from each participant at each visit. Peripheral Blood Mononuclear Cells (PBMC) were isolated from human peripheral blood of healthy volunteers. After the peripheral blood of healthy volunteers was drawn, the lymphocyte separation medium was added in a volume ratio of 1:2, and centrifuged at 1000 g × for 30 min at room temperature. After washing with PBS, erythrocyte lysate was added, and PBMCs were collected after washing with PBS again and centrifugation. The collected cells were resuspended with RPMI1640. Adjust the cell concentration to 1 × 10^6^/mL and transfer to culture flasks for culture. The medium contains 100 U/mL rhIL-2, 1 U/mL anti-CD3/CD28 antibody, 10% FBS, 1% penicillin and streptomycin mixture.

### Binding characterization of CD3-CSPG4/BiTE by ELISA and flow cytometry

To evaluate the binding capacity of CD3-CSPG4/BiTE to recombinant human CD3 (r-hCD3), recombinant human CSPG4 (r-hCSPG4), and an irrelevant control antigen (r-hCD22), each antigen was diluted to 2.5 µg/mL and coated onto 96-well plates, followed by blocking with 3% BSA. Serially diluted CD3-CSPG4/BiTE was then added, and bound BiTE was detected using an anti-His-tag-HRP conjugate, which recognizes the C-terminal His-tag on the BiTE. After washing, TMB substrate was added, and the absorbance was read at 450 nm. The absorbance values thus reflect the amount of BiTE bound to the immobilized antigen via its CSPG4-specific scFv (for r-hCSPG4) or CD3-specific scFv (for r-hCD3), with the irrelevant antigen (r-hCD22) serving as a specificity control. All measurements were performed in triplicate, and background absorbance (no BiTE) was subtracted.

A375, A2058, and SBCL-2 human melanoma cell lines, as well as T cells isolated from donor peripheral blood mononuclear cells, were collected. After washing with PBS, the cells were incubated with CD3-CSPG4/BiTE for 30 minutes and washed again. For the pre-blocked group, excess amounts of r-hCSPG4 or r-hCD3 were pre-mixed with CD3-CSPG4/BiTE before being added to the target cells for the binding assay. Then, 3 µL of anti-His-PE secondary antibody was added to each tube and incubated for 30 minutes. After a final wash with PBS, binding was assessed by flow cytometry.

### Flow cytometry

T cells were stained with CFSE for 20 min, washed, and co-cultured with A375 target cells (E:T = 10:1) in the presence of 2 µg/mL CD3-CSPG4/BiTE. After 5 days, floating T cells were harvested, washed with PBS, and analyzed on a flow cytometer using the FITC channel to assess CFSE dilution.

T cells were co-incubated with A375 cells (E:T = 10:1) and 2 µg/mL BiTE for 24 h. Harvested T cells were washed three times with PBS, resuspended in 500 µL PBS, and stained with 5 µL each of anti-human CD25-PE, CD69-APC and CD107a-APC for 30 min in the dark. After a final PBS wash, expression of activation markers was quantified by flow cytometry.

To assess CD3-CSPG4/BiTE-mediated, T cell–specific killing of A375 target cells, the targets were first harvested and labeled with eFluor670 for 20 min. Labeling was stopped by adding 3–4 volumes of complete RPMI-1640 medium followed by a 5-min incubation on ice (4 °C). T cells and labeled targets were then mixed at E:T ratios of 1:1, 10:1 and 20:1 in a final volume of 500 µL per well and co-cultured for 12 h. After co-culture, the supernatant containing non-adherent cells was collected. The remaining adherent cells were trypsinized and then pooled with the supernatant. The pooled cells were resuspended in 100 µL PBS and stained with 5 µL of 7-AAD for 30 minutes on ice in the dark. After washing with PBS, cells were acquired on a BD flow cytometer and analyzed using FlowJo v10.8.1 to quantify specific killing.

### ELISA

To quantify IFN-γ, TNF-α and IL-10 released by T cells, T cells were co-cultured with A375 targets (E:T = 10:1) plus 2 µg/mL CD3-CSPG4/BiTE; after 24 h the supernatants were collected. One-hundred-and-fifty microlitres of each supernatant was added to the corresponding well and incubated at room temperature for 2 h, followed by 200 µL biotinylated detection antibody for 70 min at room temperature. After PBS washing, 200 µL HRP-conjugated streptavidin was added and incubated for 40 min at room temperature, the wells were again PBS-washed, 200 µL substrate solution was added and incubated for 40 min at room temperature, a final PBS wash was performed, 60 µL stop solution was added and the absorbance (OD) was read on a microplate reader; cytokine concentrations were calculated from standard curves. For the in-vivo study, tumour tissue was harvested from mice, homogenised and centrifuged, and the resulting tissue fluid was assayed for IFN-γ, TNF-α and IL-2 using the identical ELISA protocol.

### In vivo studies

All in-vivo procedures were conducted in accordance with standard guidelines and protocols approved by the national and institutional animal-care committee. Female NOD/SCID mice (6–8 weeks old; Vital River, Beijing, China) were maintained under specific pathogen-free conditions at People’s Hospital of Qiannan. Mice were euthanized by placing them in a sealed chamber charged with 5% isoflurane in 1 L min^−1^ O_2_ for 30 s induction, followed by 3 min maintenance until breathing and corneal reflex ceased and cardiac arrest was confirmed. All animal studies were approved by the university’s Institutional Animal Care and Use Committee (IACUC) and conducted per national humane guidelines. A375 tumor cells were harvested, resuspended in sterile PBS, and 200 µL containing 2 × 10⁶ cells was injected subcutaneously into the right flank. Tumor growth was monitored regularly; when xenografts reached 80–100 mm³, mice with similar tumor volumes and comparable engraftment times were randomized into four groups (n = 5). Therapy lasted 7 d: each mouse received two intravenous injections of 1 × 10⁷ T cells in 200 µL PBS (days 0 and 4) and daily tail vein injections of 20 µL PBS or antibody (1 mg kg ^−1^, ≈ 20 µg per mouse). Treatment began on day 10 after tumor inoculation; tumor length and width were measured every 3 d and volume was calculated. The study was terminated on day 30, or earlier if tumor diameter exceeded 20 mm or volume exceeded 1 500 mm³. Tumors were excised to quantify infiltrating CD3 ⁺ T cells, and growth curves were constructed from tumor-weight inhibition rates. Remaining mice were followed for survival; body weight, food intake, and tumor diameter were recorded daily. Survival differences between groups were analyzed with Kaplan–Meier curves and log-rank tests.

For toxicity assessment, CD3-CSPG4/BiTE (experimental group) or PBS (control group) was administered via tail vein. Seven days later, heart, liver, spleen, lung, and kidney were harvested, fixed, sectioned, and stained with hematoxylin–eosin to evaluate histopathological changes.

### Statistical analysis

All statistical analyses were performed using GraphPad Prism 8. Data are presented as mean ± standard deviation (SD). Comparisons among multiple groups were conducted using one‑way analysis of variance (ANOVA). Mouse survival was visualized using Kaplan–Meier curves, and differences between groups were assessed by the log‑rank test. A p-value < 0.05 was considered statistically significant and is denoted as follows: ^*^p < 0.05, ^**^p < 0.01, ^***^p < 0.001, ^****^p < 0.0001. “ns” indicates no statistical significance.

## Results

### Construction, validation, and in vitro characterization of CD3-CSPG4/BiTE

In the preliminary phase of this project, high-affinity anti-CD3 and anti-CSPG4 single-chain antibodies were successfully isolated and tethered in tandem via a flexible (Gly_4_Ser)_3_ linker to generate the bispecific T-cell engager CD3-CSPG4/BiTE. Two versions of the construct were prepared (Fig 1A,B). Following IPTG induction, CD3-CSPG4/BiTE was expressed at high levels and purified by Ni-NTA affinity chromatography exploiting its C-terminal His-tag. SDS-PAGE analysis revealed a single (S1 Fig), sharp band at ≈51 kDa, confirming the purity and expected molecular mass of the recombinant protein (Fig 1C).

**Fig 1:**
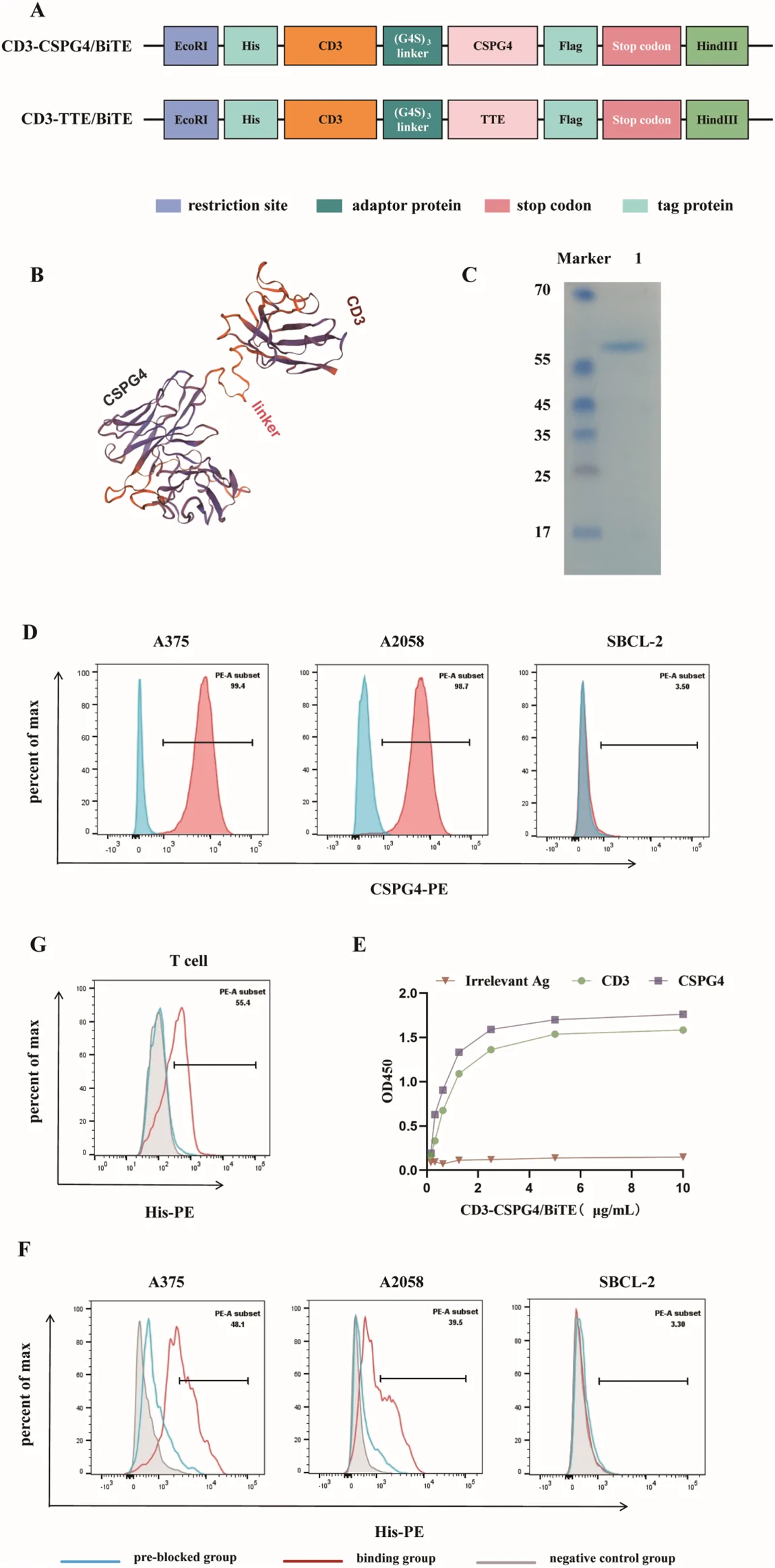
Construction and validation of CD3-CSPG4/BiTE. (A) Schematic diagram of the BiTE structures in each group. (B) Overall architecture of CD3-CSPG4/BiTE. (C) SDS-PAGE analysis of purified CD3-CSPG4/BiTE; a single, sharp band is visible at ~51 kDa. (D) Flow-cytometric quantification of CSPG4 surface expression on A375, A2058 and SBCL-2 cell lines. (E) ELISA binding profiles of CD3-CSPG4/BiTE to r-hCSPG4, r-hCD3 and an irrelevant human antigen. (F) Flow-cytometric assessment of CD3-CSPG4/BiTE binding to A375, A2058 and SBCL-2 cells. Blue histograms: cells pre-blocked with excess r-hCSPG4; red histograms: cells incubated with CD3-CSPG4/BiTE alone. (G) Binding of CD3-CSPG4/BiTE to human T cells. Blue histograms: T cells pre-blocked with excess r-hCD3; red histograms: T cells incubated with CD3-CSPG4/BiTE alone.

ELISA assays showed that CD3-CSPG4/BiTE bound to immobilized r-hCSPG4 and r-hCD3 in a dose-dependent manner, whereas no binding to an irrelevant human antigen was detected, confirming high specificity. Flow-cytometric analysis revealed high CSPG4 expression on the cutaneous melanoma lines A375 and A2058, but low expression on SBCL-2 cells(Fig 1D,E). CD3-CSPG4/BiTE selectively bound CSPG4-positive targets (A375 and A2058) and did not react with CSPG4-negative SBCL-2 cells. The binding rate to A375 (41.7 ± 4.1%) was the highest; therefore, A375 was chosen as the target cell line for all subsequent experiments(Fig 1E,F,G).

### T-cell proliferation and activation induced by CD3-CSPG4/BiTE

To monitor T-cell proliferation, CFSE-labeled T cells were divided into four groups: PBS, blocking control, CD3-TTE/BiTE, and CD3-CSPG4/BiTE (this grouping was used in all subsequent experiments). Each group was co-incubated with A375 target cells and the corresponding antibody for 5 days. Flow-cytometric analysis of CFSE dilution revealed that T cells in the CD3-CSPG4/BiTE group exhibited the highest proliferative activity (Fig 2A,B), significantly surpassing that of all other groups.

**Fig 2:**
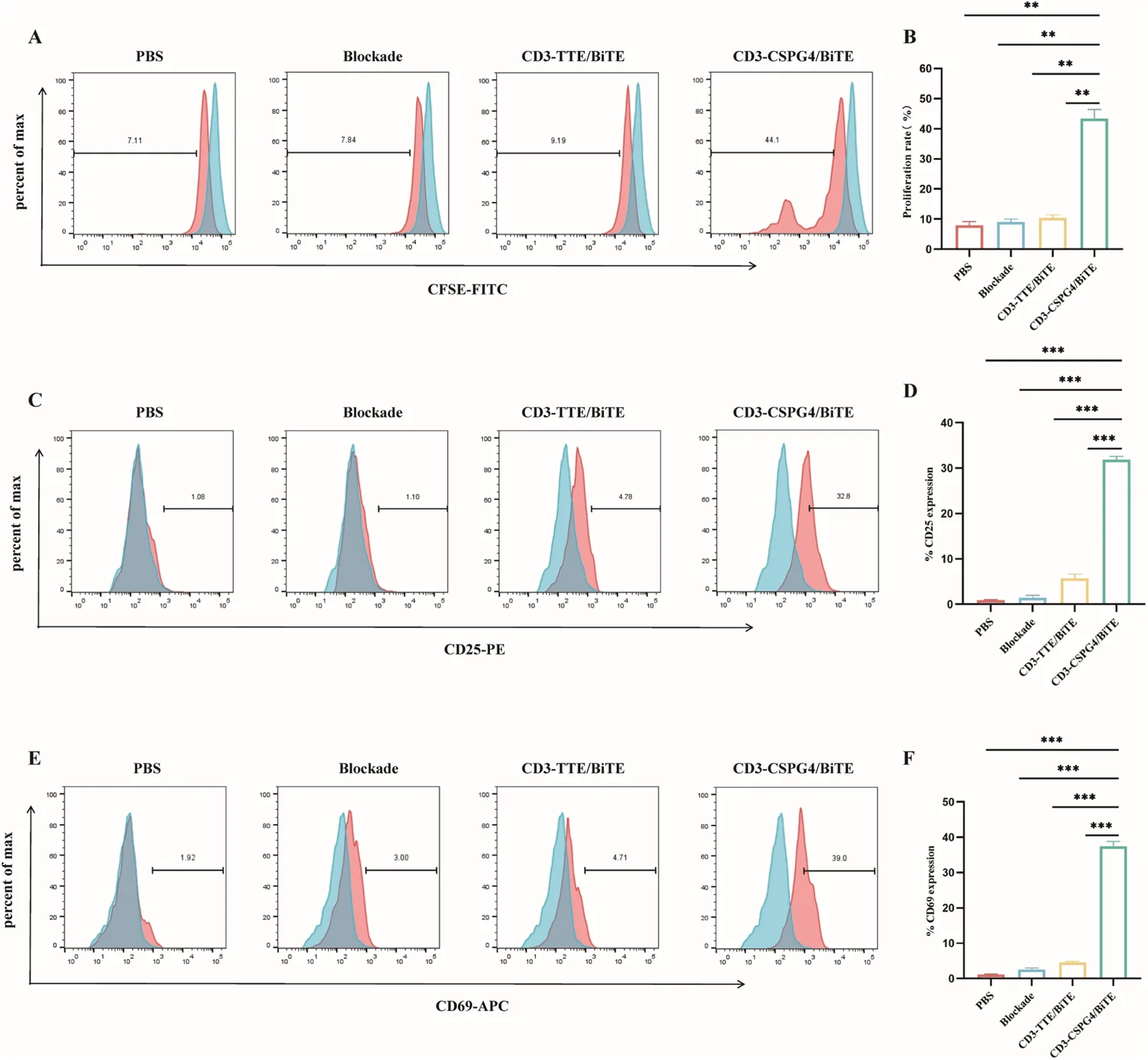
Effects of CD3-CSPG4/BiTE on T-cell proliferation and activation. (A) Representative flow-cytometric plots of CFSE dilution in T cells after 5-day co-culture with A375 cells under the indicated conditions. (B) Quantitative analysis of T-cell proliferation (n = 3). (C) CD25 expression on T cells following 24-h co-culture with A375 plus the indicated antibodies. (D) Statistical summary of CD25 levels. (E) CD69 expression on T cells under the same conditions as in (C). (F) Statistical summary of CD69 levels. The data are shown with the mean ± SD of three independent experiments. ^*^P < 0.05,^**^P < 0.01,^***^P < 0.001, represent the difference is statistically significant. ns *P* > 0.05, represented the difference was no statistically significant.

To evaluate the activating effect of CD3‑CSPG4/BiTE on T cells, CD3‑CSPG4/BiTE and the respective control groups were co‑cultured with A375 cells for 24 hours. T cells were then collected from each group and analyzed by flow cytometry. The results showed that after stimulation with A375 combined with CD3‑CSPG4/BiTE, the surface expression levels of CD25 (Fig 2C,D) and CD69 (Fig 2E,F) on T cells were significantly higher than those in the other control groups(^***^P < 0.001), indicating that CD3‑CSPG4/BiTE effectively promotes T‑cell activation.

### Cytotoxicity assays against tumor cell lines with CD3-CSPG4/BiTE

To further evaluate the cytotoxic specificity and potency of CD3-CSPG4/BiTE in vitro, CSPG4-positive A375 cells were employed as targets and co-cultured with T cells at E:T ratios of 1:1, 10:1 and 20:1. The percentage of double-positive target cells was then quantified by flow cytometry to calculate killing efficiency. CD3-CSPG4/BiTE directed robust, ratio-dependent lysis of A375 cells; cytotoxicity rose progressively as the E:T ratio increased (Fig 3A). To define the dose–response relationship (E:T = 10:1), five serial concentrations of CD3-CSPG4/BiTE were tested (Fig 3B). At all doses, killing of CSPG4-high A375 target cells rose sharply with increasing BiTE concentration and plateaued at ≥10 µg/mL, indicating potent, antigen-specific cytotoxicity, whereas the other control BiTEs showed no significant killing of A375 target cells.

**Fig 3:**
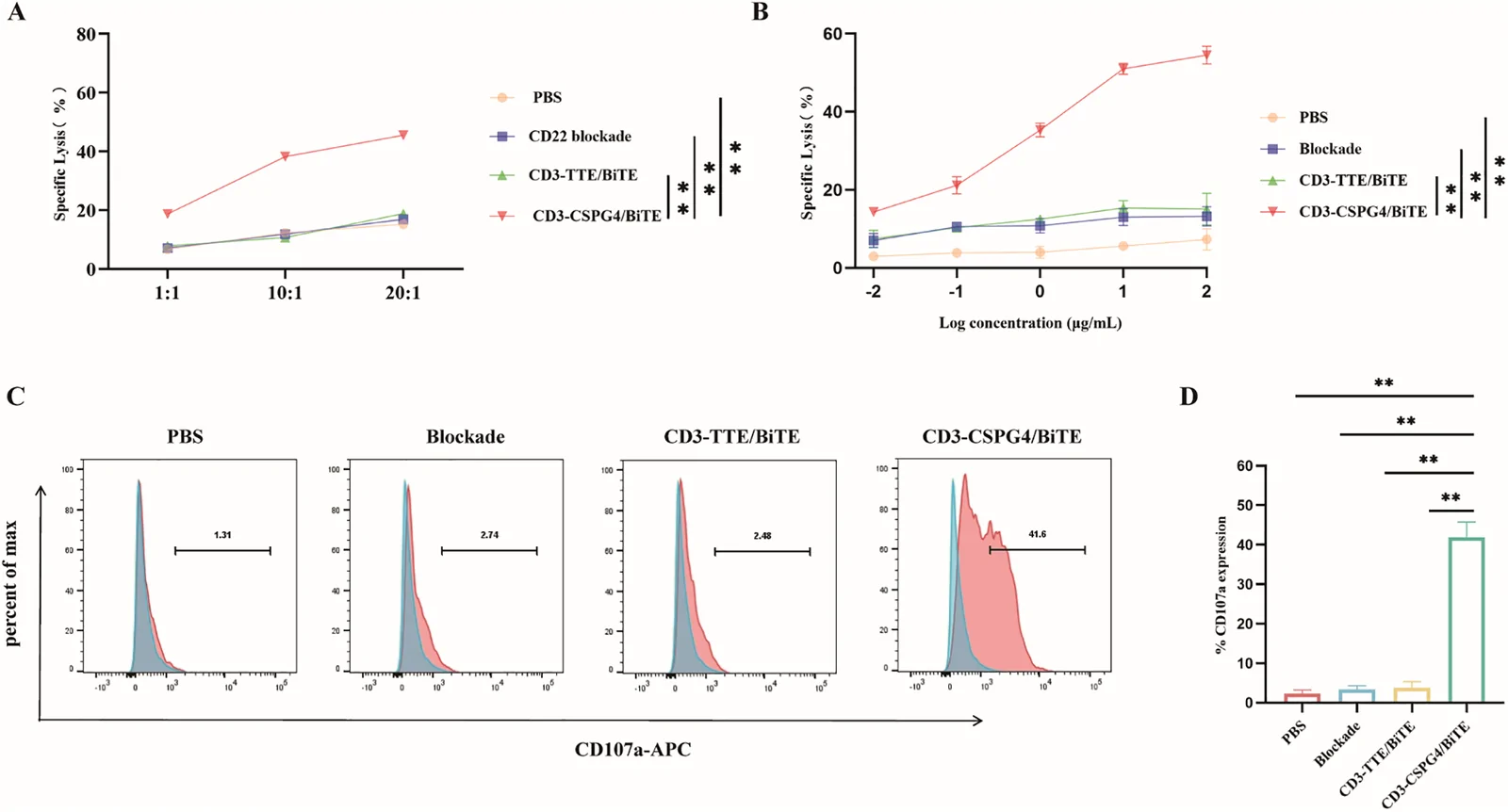
CD3-CSPG4/BiTE-mediated T-cell cytotoxicity against target cells. (A) Dose-dependent killing of A375 cells by T cells in the presence of escalating concentrations of CD3-CSPG4/BiTE or control BiTE (E:T = 10:1). (B) Cytotoxicity mediated by CD3-CSPG4/BiTE at varying E:T ratios (1:1, 10:1, 20:1). (C) Flow-cytometric histograms of CD107a surface expression on T cells after 6 h co-culture with A375 plus the indicated antibodies. (D) Quantitative analysis of CD107a levels (mean ± SD, n = 3).he data are shown with the mean ± SD of three independent experiments. ^*^P < 0.05,^**^P < 0.01,^***^P < 0.001, represent the difference is statistically significant. ns *P* > 0.05, represented the difference was no statistically significant.

To examine the degranulation response, T cells were co-incubated with A375 target cells in the presence of either CD3-CSPG4/BiTE, control BiTE, or PBS for 6 h, and surface CD107a was measured by flow cytometry. CD107a expression was markedly higher in the CD3-CSPG4/BiTE group than in any other condition (Fig 3C, D), indicating enhanced T-cell degranulation.

### Impact of CD3-CSPG4/BiTE on cytokine secretion by T cells

During activation, differentiation, and immune regulation, T cells secrete a variety of cytokines. To quantify these mediators, T cells were co‑cultured with A375 target cells in the presence of the indicated BiTE antibodies, and cytokine levels were measured by ELISA. TNF‑α (Fig 4A) secretion was highest in the CD3‑CSPG4/BiTE group and was significantly elevated compared with all BiTE‑naïve controls (*P* < 0.01), an identical pattern was observed for IFN‑γ (Fig 4B). In contrast, no significant differences in IL‑10 (Fig 4C) or IL‑6 (Fig 4D) levels were detected between the CD3‑CSPG4/BiTE group and any control group (*P* > 0.05).

**Fig 4:**
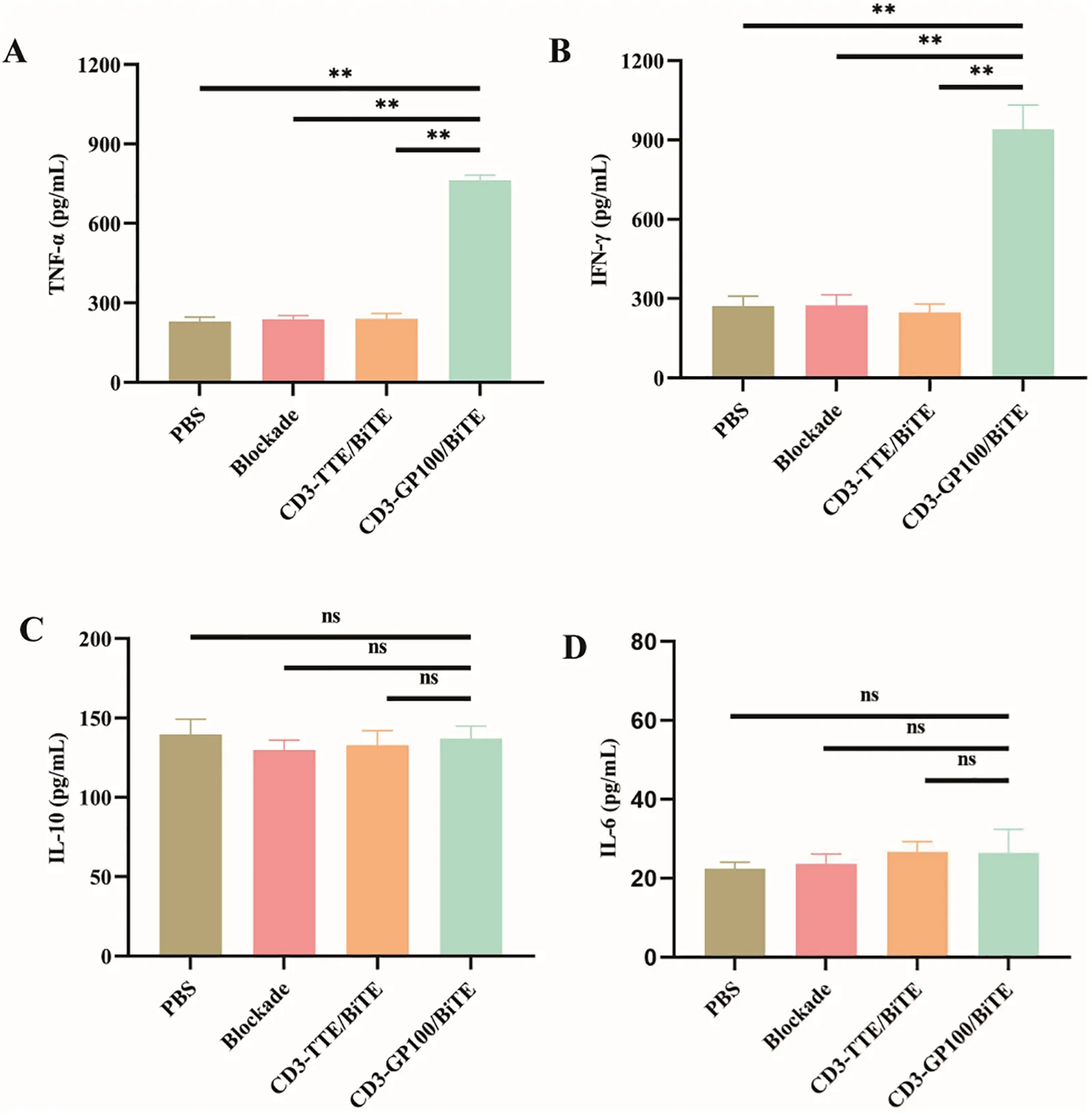
Cytokine secretion profiles of T cells after co-culture with A375 melanoma cells in the presence of BiTE antibodies. T cells were co-cultured with A375 target cells with or without the indicated BiTE antibodies. Supernatants were collected and cytokine levels were measured by ELISA. (A) TNF-α secretion was highest in the CD3-CSPG4/BiTE group and was significantly elevated compared with all BiTE-naïve controls (P < 0.01). (B) IFN-γ showed an identical pattern, with significantly higher levels in the CD3‑CSPG4/BiTE group (^**^P < 0.01). (C, D) No significant differences in IL-10 (C) or IL-6 (D) levels were detected between the CD3-CSPG4/BiTE group and any control group (P > 0.05). The data are shown with the mean ± SD of three independent experiments. ^*^P < 0.05,^**^P < 0.01, represent the difference is statistically significant. ns *P* > 0.05, represented the difference was no statistically significant. Statistical significance was determined by one‑way ANOVA followed by appropriate post-hoctest.

### In vivo studies

To evaluate the non‑specific biological toxicity of the CD3‑CSPG4/BiTE protein itself, mice were administered CD3‑CSPG4/BiTE (experimental group) or PBS (control group) via tail vein injection daily for 7 days. Histopathological examination revealed no significant abnormalities in the heart, liver, spleen, lung, or kidney of BiTE‑treated mice compared with PBS controls (Fig 5). These observations preliminarily indicate that the protein preparation is of good quality and has a low intrinsic toxicity risk.

**Fig 5:**
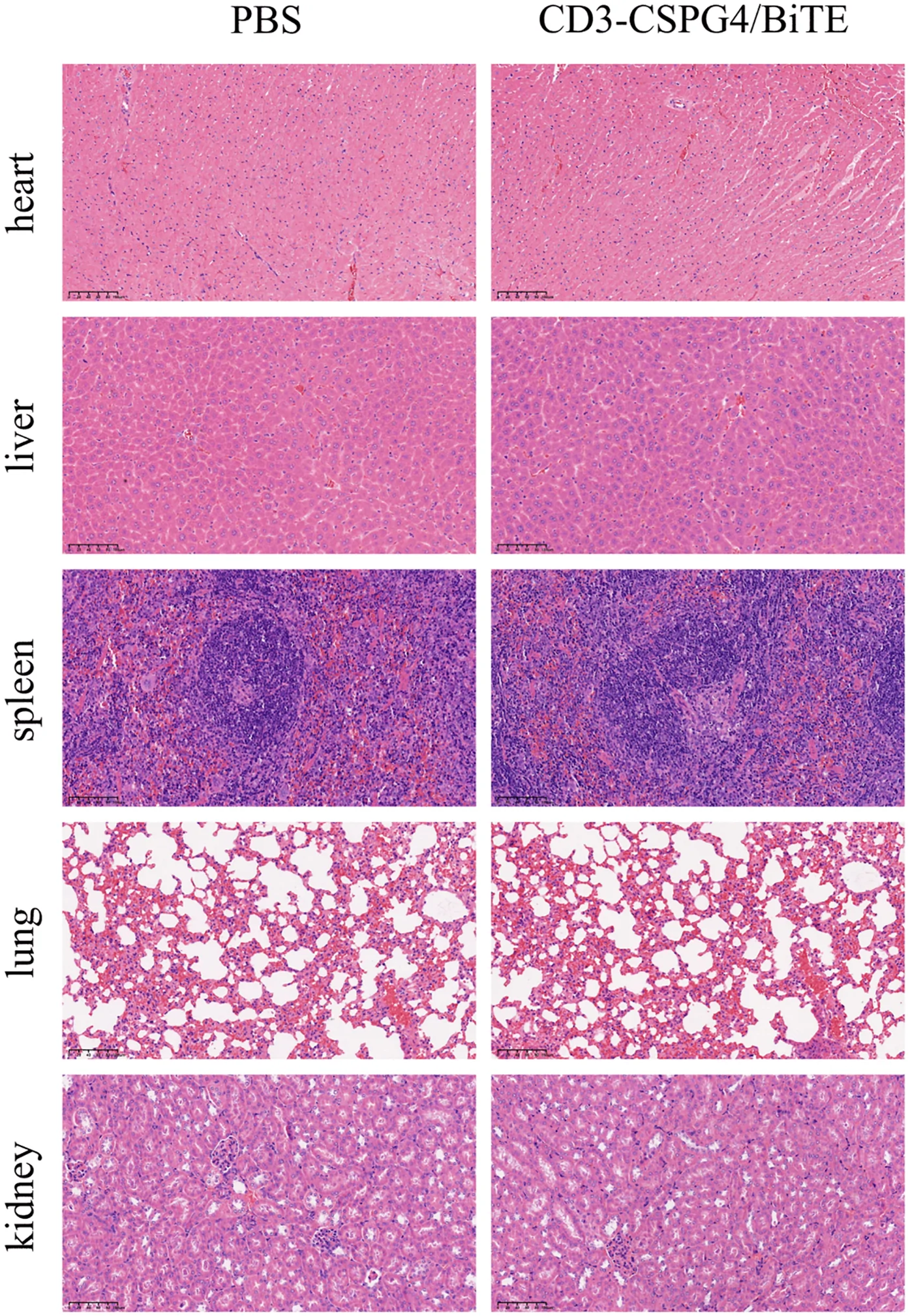
HE staining results of the heart, liver, spleen, lung, and kidney in normal BALB/c mice.

To further evaluate the in vivo antitumor activity of T cells redirected by CD3-CSPG4/BiTE, a subcutaneous A375 xenograft model was established in NOD/SCID mice using the CSPG4-high melanoma cell line A375. Mice were treated with a combination of CD3-CSPG4/BiTE and human T cells (Fig 6A). A375 cells were injected subcutaneously on day 0, and palpable nodules developed at the injection sites approximately 7 days later. Tumor take was confirmed by manual palpation in all mice, and the animals remained in good general condition. Compared with PBS, CD3-TTE/BiTE, and CSPG4-blockade controls, treatment with CD3-CSPG4/BiTE markedly reduced both tumor volume and final tumor weight, exhibiting the strongest inhibition of tumor growth and the greatest prolongation of survival (Fig 6B–E).

**Fig 6:**
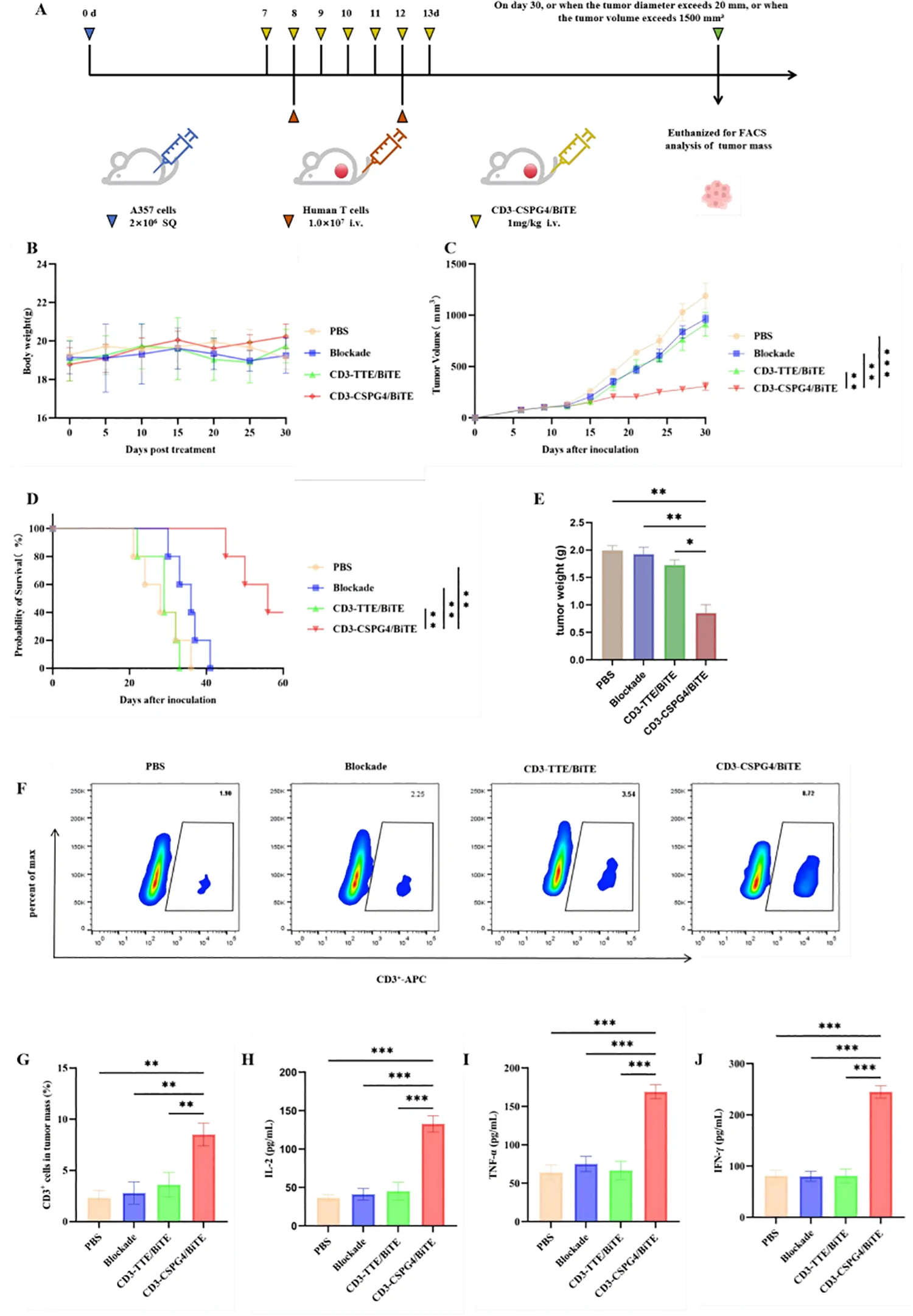
CD3-CSPG4/BiTE redirects human T cells to suppress A375 melanoma growth in vivo and remodels the tumor immune microenvironment. (A) Schematic illustration of the experimental design. NOD/SCID mice were implanted subcutaneously with A375 human melanoma cells (day 0), followed by adoptive transfer of human T cells together with the indicated treatments. (B-E) Antitumor efficacy of CD3-CSPG4/BiTE. Compared with PBS, CD3-TTE/BiTE, and CSPG4-blockade controls, CD3-CSPG4/BiTE treatment significantly reduced tumor volume over time (B), final tumor weight (C), and prolonged mouse survival (D, E). Data are shown as mean ± SEM (n = 5 mice per group). (F, G) Intratumoral T-cell infiltration. Tumors were harvested at the endpoint, dissociated into single-cell suspensions, and stained for human CD3. Representative flow cytometry plots (F) and quantification (G) show that CD3-CSPG4/BiTE treatment resulted in a significantly higher frequency of CD3 ⁺ T cells within tumors compared with all control groups. (H-J) Cytokine production in the tumor microenvironment. Tumor homogenates were analyzed by ELISA. Levels of IL-2 (H), TNF-α (I), and IFN-γ (J) were markedly elevated in the CD3‑CSPG4/BiTE group relative to controls. Statistical significance was determined by one‑way ANOVA followed by Tukey’s post‑hoc test. ^*^p < 0.05, ^**^p < 0.01, ^***^p < 0.001. All experiments were repeated at least twice with similar results. Data are presented as mean ± SD.

To evaluate the therapeutic efficacy, immune‑microenvironment alterations, and safety of CD3‑CSPG4/BiTE in vivo, tumors from A375‑bearing mice were excised after treatment completion, processed into single‑cell suspensions, and stained with anti‑hCD3‑APC for flow cytometry. CD3 ⁺ T cells were detected in all groups, but their frequency was substantially higher in tumors from CD3‑CSPG4/BiTE‑treated mice than in any control group (Fig 6F,G). This robust in situ expansion of CD3 ⁺ T cells, driven by concurrent engagement of tumor cells and the BiTE, likely underlies the superior tumor‑clearing capacity of CD3‑CSPG4/BiTE (^*^p < 0.05, ^**^p < 0.01). Furthermore, ELISA analysis of tumor homogenates revealed that, compared with all control groups, mice receiving CD3‑CSPG4/BiTE‑mediated T‑cell therapy showed significantly elevated secretion of IL‑2 (Fig 6H), TNF‑α (Fig 6I), and IFN‑γ (Fig 6J), indicating that the BiTE markedly enhances local inflammatory cytokine production (^***^p < 0.001). Collectively, these data demonstrate that CD3‑CSPG4/BiTE exerts potent anti‑melanoma activity in vivo.

## Discussion

Malignant melanoma (MM) is highly invasive and metastasizes early; once conventional therapies fail, the median survival is less than one year. Here we designed and validated a novel bispecific T cell engager, CD3-CSPG4/BiTE, that targets CSPG4 — an antigen over-expressed on MM cells but scarcely on normal tissues. Both in vitro and in vivo studies demonstrate that this construct robustly activates T cells, efficiently eliminates CSPG4 ⁺ tumors, and maintains an acceptable safety profile, offering a promising new strategy for MM therapy.

MM predominantly arises on acral skin (e.g., soles, fingertips) and mucosal sites (e.g., nasal and oropharyngeal mucosa). Because it readily metastasizes, it remains the cutaneous neoplasm with the poorest prognosis and one of the highest mortality rates [18,19]. Conventional surgery, chemotherapy, radiotherapy or their combinations have achieved unsatisfactory outcomes in advanced or metastatic MM, so comprehensive antitumor strategies are urgently needed in current clinical practice. Over the past decade, immune checkpoint inhibitors (e.g., anti-PD-1/PD-L1 and anti-CTLA-4 antibodies) and BRAF/MEK-targeted therapies have significantly improved clinical outcomes in patients with advanced melanoma. However, approximately 50% of patients do not respond to immune checkpoint inhibitors, and most patients who initially respond to BRAF/MEK inhibitors eventually acquire resistance within a median of about 12 months, leading to disease progression. In this context, bispecific T‑cell engagers (BiTEs), as an MHC‑I‑independent immunotherapy strategy, offer a new approach to overcome the limitations of current therapies [20–22]. CSPG4 is highly expressed on the surface of malignant tumor cells but is low or restricted in non‑malignant tissues. Accumulating evidence indicates that it also acts as an oncogene, driving tumor progression and metastasis through multiple mechanisms. Given the inherent immunogenicity of melanoma, CSPG4‑targeted therapies could enhance immune surveillance, thereby promoting the selective elimination of tumor cells. Monoclonal antibodies, CAR T, NKT, and γ/δ T cell-engineered constructs, antibody–drug conjugates, and anti‑idiotypic or linear peptide vaccines have all shown preclinical and early clinical efficacy against melanoma both in vitro and in vivo. However, few of these strategies have advanced to later‑stage human trials, and none are currently being evaluated in patients [23–25]. A multifaceted strategy is required to translate the therapeutic potential of CSPG4-targeted agents into clinical benefit.Based on this concept, the present study designed a CD3‑CSPG4 bispecific T‑cell engager. The mechanism of action of CSPG4‑targeting BiTE is fundamentally different from that of existing melanoma immunotherapies and targeted therapies. Immune checkpoint inhibitors relieve T cell exhaustion by blocking the PD-1/PD-L1 signal, but their efficacy depends on the effective presentation of antigenic peptides by tumor cells through MHC-I molecules and the presence of functional T cells within the tumor microenvironment [26,27]. BRAF/MEK inhibitors directly target the MAPK signaling pathway and can rapidly induce tumor regression in patients with BRAFV600 mutations, but acquired resistance is almost inevitable, mainly through MAPK pathway reactivation and compensatory bypass signaling [28,29]. In contrast, the CSPG4-targeting BiTE directly redirects T cells to CSPG4-positive tumor cells in an MHC‑independent manner, triggering specific T cell killing activity. This mechanism intrinsically circumvents immune escape caused by MHC-I downregulation and is independent of MAPK pathway activation status, thus offering the potential to overcome resistance to current therapies [30].

In vitro, CD3-CSPG4/BiTE markedly enhanced T-cell proliferation. Upon co-culture with A375 cells, T cells exhibited significantly upregulated activation markers CD25 and CD69. Moreover, the BiTE construct potently induced specific lysis of A375 targets, confirming its robust antitumor activity in vitro. ELISA further revealed that CD3-CSPG4/BiTE stimulation elevated IFN-γ and TNF-α secretion, which reinforces T-cell activation and expansion, whereas IL-10 and IL-6 release remained unchanged. Notably, unchanged IL-6 is advantageous, since this pro-inflammatory cytokine is central to cytokine-storm pathophysiology; through JAK/STAT signaling, excessive IL-6 drives systemic inflammation culminating in multi-organ dysfunction and failure [31–33]. IL-10 is a pleiotropic cytokine that primarily limits inflammatory responses; however, during a cytokine storm its excessive production can overstimulate the immune system and drive the release of additional cytokines, thereby amplifying the storm [34,35]. The CD3-CSPG4/BiTE construct showed a favorable safety profile in this preliminary assessment; however, the risk of cytokine release syndrome (CRS) requires further dedicated in vivo pharmacokinetic and toxicity studies, including measurement of serum cytokines.

Consistent with the in vitro observations, in vivo administration of CD3-CSPG4/BiTE produced no evident histopathological changes in major murine organs. Throughout the A375 subcutaneous xenograft study, body weights of CD3-CSPG4/BiTE-treated mice remained comparable to those of control animals. In this A375 model, CD3-CSPG4/BiTE significantly prolonged survival: median survival increased from 33 days in the PBS group to 49 days in the BiTE group (*p* < 0.01), and two of five mice remained tumor-bearing but alive at the study endpoint (day 60), suggesting the establishment of long-term immune memory. Meanwhile, no significant weight loss was observed. Consistent with the survival benefit, tumor volume in the CD3-CSPG4/BiTE group on day 30 was only 317 ± 38 mm³—less than 30% of that in control mice—indicating that BiTE-redirected T-cell killing rapidly curtailed solid tumor growth. Flow cytometry further revealed that intratumoral CD3 ⁺ T cells accounted for 8.5% of live cells, an approximately 3-fold increase over controls (3.2%). Functionally, ELISA of tumor lysates showed IFN-γ, TNF-α, and IL-2 levels elevated approximately 3-fold, 2-fold, and 4-fold, respectively, demonstrating that the BiTE remodels the tumor microenvironment. Notably, efficacy correlated positively with both T-cell abundance and functional status within tumors, offering guidance for optimal dosing schedules in forthcoming clinical applications.

CD3-CSPG4/BiTE bridges autologous T cells and melanoma cells by simultaneously engaging CD3 on T cells and the melanoma-associated antigen CSPG4, thereby mediating an immunological synapse between the two cell types. This leads to rapid induction of IFN-γ, TNF-α, and IL-2, markedly enhances intratumoral infiltration of cytotoxic T lymphocytes, and prolongs the survival of tumor‑bearing animals in a mouse subcutaneous xenograft melanoma model. Although the objective response rate, infusion‑related toxicities, and antigen escape of this therapy still require further optimization, CD3-CSPG4/BiTE overall represents a T-cell redirecting platform that is independent of TCR affinity, modularly designable, and readily applicable. Its mechanistic and safety data provide a solid experimental foundation for subsequent clinical translation and the continued iteration of bispecific antibody technologies.

## Supporting information

S1 Fig Original uncropped and unadjusted SDS-PAGE gel images. (JPG)
